# The cumulative duration of bispectral index less than 40 concurrent with hypotension is associated with 90-day postoperative mortality: a retrospective study

**DOI:** 10.1186/s12871-020-01122-7

**Published:** 2020-08-14

**Authors:** Soohyuk Yoon, Seokha Yoo, Min Hur, Sun-Kyung Park, Hyung-Chul Lee, Chul-Woo Jung, Jae-Hyon Bahk, Jin-Tae Kim

**Affiliations:** 1 Department of Anesthesiology and Pain Medicine, Seoul National University College of Medicine, Seoul National University Hospital, 101 Daehak-ro, Jongno-gu, Seoul, 03080 Republic of Korea; 2 Department of Anesthesiology and Pain Medicine, Ajou University Hospital, 164 World cup-ro, Yeongtong-gu, Suwon-si, Gyeonggi-do 16499 Republic of Korea

**Keywords:** Bispectral index, Intraoperative hypotension, Postoperative mortality, Major abdominal surgery

## Abstract

**Background:**

The relationship between intraoperative low bispectral index (BIS) values and poor clinical outcomes has been controversial. Intraoperative hypotension is associated with postoperative complication. The purpose of this study was to investigate the influence of intraoperative low BIS values and hypotension on postoperative mortality in patients undergoing major abdominal surgery.

**Methods:**

This retrospective study analyzed 1862 cases of general anesthesia. We collected the cumulative time of BIS values below 20 and 40 as well as electroencephalographic suppression and documented the incidences in which these states were maintained for at least 5 min. Durations of intraoperative mean arterial pressures (MAP) less than 50 mmHg were also recorded. Multivariable logistic regression was used to evaluate the association between suspected risk factors and postoperative mortality.

**Results:**

Ninety-day mortality and 180-day mortality were 1.5 and 3.2% respectively. The cumulative time in minutes for BIS values falling below 40 coupled with MAP falling below 50 mmHg was associated with 90-day mortality (odds ratio, 1.26; 95% confidence interval, 1.04–1.53; *P* = .019). We found no association between BIS related values and 180-day mortality.

**Conclusions:**

The cumulative duration of BIS values less than 40 concurrent with MAP less than 50 mmHg was associated with 90-day postoperative mortality, not 180-day postoperative mortality.

## Background

Monitoring anesthesia depth is essential for providing optimal anesthesia as it enables the maintenance of adequate anesthesia level [1]. The bispectral index (BIS) monitor can reduce the risk of intraoperative awareness as well as facilitate faster recovery after general anesthesia by enabling the anesthesiologists to appropriately adjust the anesthetic dose [2, 3]. Recently, there has been a growing interest in how the depth of anesthesia monitored using BIS affects postoperative outcomes.

Several studies have suggested an association between low BIS value (< 40 or 45) and postoperative mortality [4–6]. However, data on a definite relationship between these remain inconclusive considering other studies [7, 8]. Sesller and colleagues first proposed that the low mean arterial pressure (MAP) during low minimum alveolar concentration (MAC) of inhalation anesthetics combined with low BIS value was a predictor of mortality, [9] followed by conflicting results [10, 11]. Another study showed an association between intraoperative electroencephalographic (EEG) suppression and postoperative mortality, only when EEG suppression was concomitant with low MAP (< 55 mmHg) [12].

Furthermore, other studies revealed that prolonged concurrent double low time of BIS and MAP was associated with higher mortality. Maheshwari and colleagues focused on 30-day mortality after cardiac surgery and they set the cutoff value of double low by calculating time-weighted average of BIS and MAP, which were < 43 and < 75 mmHg respectively [13]. Meanwhile, other prospective study used the thresholds of 75 mmHg for MAP and 45 for BIS and involved all surgical specialties of noncardiac surgery to investigate 90-day mortality [14].

Intraoperative low BIS values and hypotension can have an influence on postoperative mortality. However, it remains unclear whether low BIS values concomitant with hypotension can affect intermediate to long term mortality considering the type of surgery and definition of double low [12, 13]. The relationship between the cumulative duration of low BIS value or EEG suppression and poor clinical outcomes also remains to be determined. The primary goal of this study was to determine whether intraoperative low BIS value (< 40 or 20), EEG suppression and low BIS value coupled with hypotension are associated with postoperative mortality in patients who underwent major abdominal surgery.

## Methods

This manuscript adheres to the applicable STROBE (Strengthening the Reporting of Observational Studies in Epidemiology) guidelines.

### Patient population

The intraoperative data used in this study were obtained from the “Registry Construction of Intraoperative Vital Signs and Clinical Information in Surgical Patients” study (H-1408-101-605, NCT02914444), which was designed to store intraoperative time-synchronized data from multiple anesthesia devices including patient monitors, anesthesia machines, BIS monitors, cardiac output monitors, and target-controlled infusion pumps by use of the ‘Vital Recorder’ (VitalDB team, Seoul, Korea) program. Using this registry, we could obtain complete intraoperative data (BIS-derived values, MAPs, and anesthetic concentrations).

Data collected for this study came from adult patients who underwent surgeries at Seoul National University Hospital between August 2016 and June 2017 under general anesthesia with BIS monitoring (BIS Vista, Covidien, Dublin, Ireland). The surgical procedures performed included abdominal surgeries on the gastrointestinal tract, liver, biliary tract, and pancreas. Data from the following cases were excluded: patients under 18 years old, cases with missing BIS value and MAP data more than 60 s, anesthesia times of less than 60 min, incomplete data on mortality, and reoperations during the period of analysis.

### Data collection

Vital sign data and clinical information pertaining to the cases were retrospectively analyzed. The data included patient’s diagnosis, age, sex, height, weight, type of operation, type and duration of anesthesia, propofol (Fresofol MCT inj 2%, Fresenius Kabi) concentration, MAC of volatile anesthetics, intraoperative BIS values, and arterial blood pressure. When MAP was less than 20 mmHg or greater than 200 mmHg, and when BIS was 0, these values were regarded as missing values.

To investigate the relationship between the duration of low BIS value maintenance and postoperative outcomes, we estimated the cumulative time in which BIS values were less than 20 or 40 and designated these as “bis20_dur” and “bis40_dur” respectively. To calculate total time of EEG suppression, designated as “eegsup_dur”, we used a suppression ratio. The suppression ratio is the percentage of time over the last 63-s period in which the signal is considered to be in the suppressed state. As an example, a suppression ratio of 40 would mean “isoelectric over 40% of the last 63 seconds”. After documenting suppression ratios at every second during anesthesia, we estimated the total time during which a patient’s EEG was suppressed by summing each case’s fractional suppression ratios applying a method used previously [12]. Lastly, we divided the sum by 60 to convert seconds to minutes and then by 100 to make percentages absolute numbers. To investigate the effects of short duration of brain suppression on clinical outcomes, we looked at the incidence in which cumulative time of BIS values less than 20 or 40 and EEG suppression lasted more than 5 min (bis20_5min, bis40_5min, and eegsup_5min respectively). To evaluate the influence of hypotension, we estimated the total time that MAP was lower than 50 mmHg (map50_dur) considering previous study [15]. We also calculated the cumulative time that MAP was less than 50 mmHg and BIS values were less than 20 or 40 simultaneously (bis20map50_dur, bis40map50_dur).

Potential clinical risk factors of postoperative mortality and delirium were determined *in priori* by clinical relevance or significance following a previous study [12]. We reviewed electronic medical records to retrieve the variables related to postoperative mortality and delirium. They included American Society of Anesthesiologists (ASA) physical status, past medical histories including the presence of aortic stenosis, congestive heart failure, coronary artery disease, hypertension, peripheral vascular occlusive disease, dysrhythmia, chronic obstructive pulmonary disease, pulmonary hypertension, stroke, malignancy, diabetes mellitus, sleep apnea, social history of smoking and drinking, and preoperative laboratory test results including hemoglobin (g/dL) and albumin (g/dL).

### Postoperative outcome

Mortality data were obtained from the Korean Ministry of the Interior and Safety using the resident registration number for each patient in February 2018. In this process, every piece of personal information collected was encrypted so as to maintain patient confidentiality. Mortality data were divided into 90-day postoperative mortality and 180-day mortality to compare early-to-intermediate term and intermediate-to-long term outcomes [16].

### Statistical analysis

Normality of continuous variables was verified with Kolmogorov–Smirnov test. In the univariable analysis, each variable of the data was analyzed by binary logistic regression in ‘enter’ method as an independent variable of postoperative mortality. Variables yielding *P*-values under 0.2 in the univariable analysis were selected as potential risk factors for multivariable analysis.

After the univariable analysis, we confirmed multicollinearity by calculating VIF (variance inflation factor) values of the potential risk factors and used value of 10 as the VIF threshold. Considering the multicollinearity, we used 2-step multivariable analysis to select more reliable variables. In the first step, among the selected risk factors from univariable analysis, variable considered to have a multicollinearity was separately included in binary logistic regression with ‘enter’ method after excluding possibly related variables. In this step, we removed potential BIS or MAP derived variables not yielding *P*-values under 0.05. In second step, selected BIS or MAP derived variables in first step and other potential risk factors not related to BIS or MAP in univariable analysis were included in final multivariable logistic regression analysis in ‘backward LR’ method. Variables remaining in the final logistic regression model were regarded as significant risk factors. The Hosmer-Lemeshow goodness-of-fit test was used to compare the estimate with the observed likelihood of outcomes.

To compare anesthetic concentration and double low duration between patients with and without adverse outcome, we used Student *t* test or Mann-Whitney U test, as appropriate. All statistical analyses were performed using SPSS software version 23 (IBM Corp., Armonk, New York, USA) and RStudio software version 1.2 (R studio, Boston, Massachusetts, USA).

## Results

**Fig. 1:**
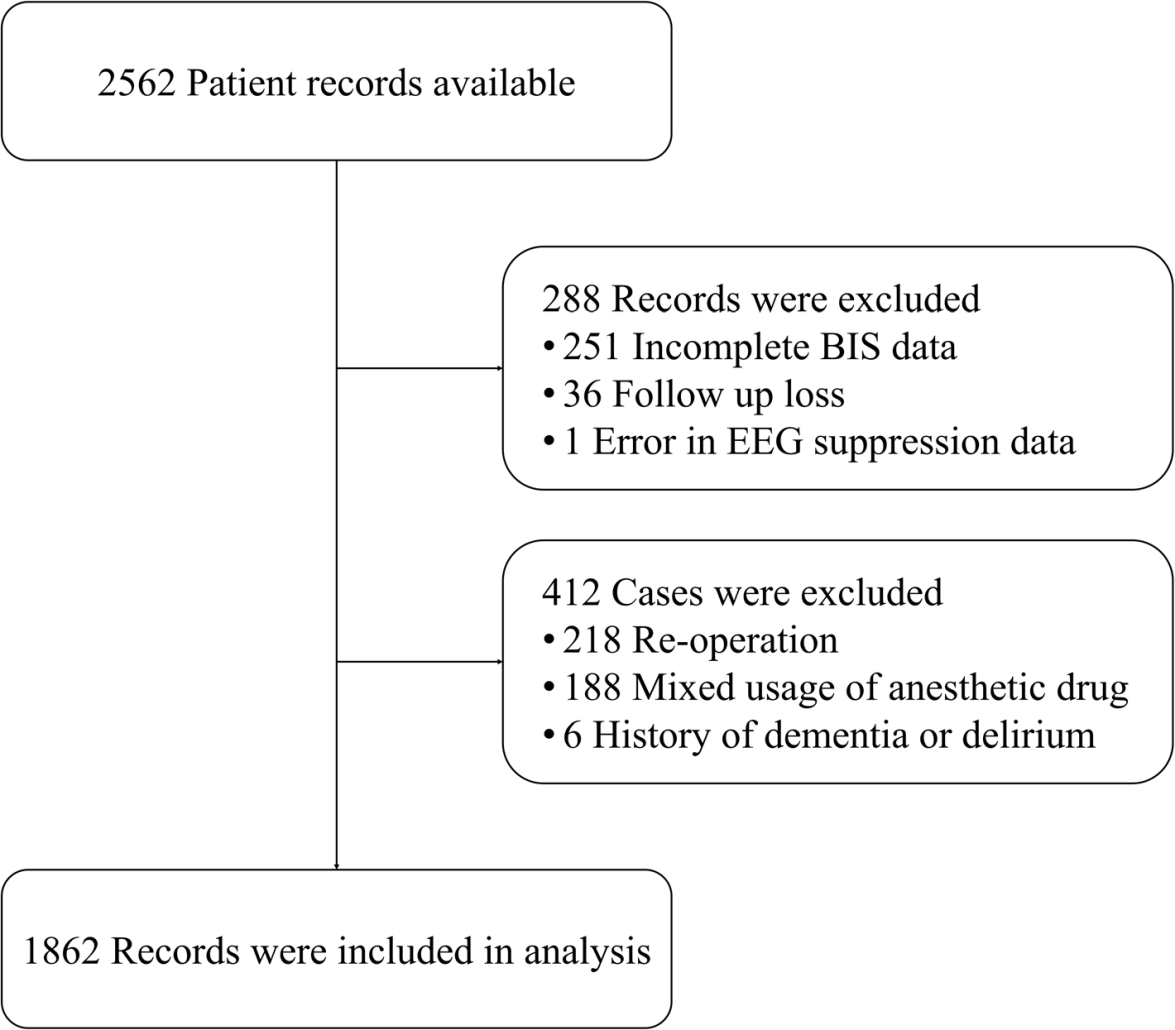
CONSORT flowchart: only remained cases after exlcusion were included for statistical analysis. BIS, bispectral index; EEG, electroencephalogram

The total number of cases during the period in the H-1408-101-605 registry were 6423 and we included 2562 cases according to surgical procedure. After applying exclusion criteria, a total of 1862 records were included. Causes of exclusion are described in a CONSORT flowchart (Fig. 1).

In the study cohort, 90-day postoperative mortality and 180-day postoperative mortality were 1.5 and 3.2%, respectively. Demographics and basic patient characteristics, specifics of the operation and anesthesia, numeric details of the BIS-derived variables and other covariates are summarized with their mean and standard deviation (SD) or number with percentage (%) in Table 1.

**Table 1:** Characteristics of cohort

Variables	All patients (*n* = 1862)
Age (year)	63.1 (19–91)
Male sex	1088 (58.4%)
Body mass index (kg/m^2^)	23.4 (3.5)
Category of surgical procedures	
Stomach	492 (26.4%)
Colorectal	719 (38.6%)
Hepatic	154 (8.3%)
Biliary-pancreas	497 (26.7%)
Type of anesthesia	
Total intravenous anesthesia	865 (46.5%)
Volatile agent	997 (53.5%)
Duration of anesthesia (min)	196.6 (104.4)
BIS derived variables	
bis40_dur (min)	70.5 (73.2)
bis40_5min	1701 (91.4%)
bis20_dur (min)	0.5 (4.2)
bis20_5min	35 (1.9%)
eegsup_dur (min)	1.6 (6.6)
eegsup_5min	134 (7.2%)
bis40map50_dur (min)	0.34 (0.92)
bis20map50_dur (min)	0.01 (0.11)
map50_dur (min)	0.8 (1.7)
Past medical history	
Aortic stenosis	10 (0.5%)
Congestive heart failure	6 (0.3%)
Coronary artery disease	98 (5.3%)
Hypertension	706 (37.9%)
Peripheral vascular occlusive disease	6 (0.3%)
Dysrhythmia	61 (3.3%)
Chronic obstructive pulmonary disease	46 (2.5%)
Pulmonary hypertension	7 (0.4%)
Stroke	70 (3.8%)
Malignancy	1368 (73.5%)
Diabetes	398 (21.4%)
Sleep apnea	3 (0.2%)
Social history	
Current smoker	255 (13.7%)
Regular alcohol ingestion	449 (24.1%)
ASA classification	
I	456 (24.5%)
II	1181 (63.4%)
III	221 (11.9%)
IV	4 (0.2%)
Laboratory tests	
Hemoglobin (g/dL)	10.5 (3.7)
Albumin (g/dL)	3.7 (1.0)

Continuous variables are presented with their mean (standard deviation) except age [mean (range)], and categorical variables are presented with their number (percentage)

Abbreviations: *BIS* Bispectral index; bis40_dur, cumulative time in which BIS < 40; bis40_5min, incidence in which cumulative time of BIS < 40 lasted > 5 min; bis20_dur, cumulative time in which BIS < 20; bis20_5min, incidence in which cumulative time of BIS < 20 lasted > 5 min; *EEG* Electroencephalogram; eegsup_dur, cumulative time in which patient’s EEG was suppressed; eegsup_5min, incidence in which cumulative time of EEG suppression lasted > 5 min; *MAP* Mean arterial pressure; bis40map50_dur, cumulative time that BIS < 40 and MAP < 50 mmHg simultaneously; bis20map50_dur, cumulative time that BIS < 20 and MAP < 50 mmHg simultaneously; map50_dur, cumulative time that MAP < 50 mmHg; *ASA* The American Society of Anesthesiologists physical status

### 90-day mortality

In univariable analysis, age, male sex, dysrhythmia, chronic obstructive pulmonary disease, pulmonary hypertension, malignancy, diabetes, ASA classification, hemoglobin levels, albumin levels, map50_dur and bis40map50_dur were found to be potential risk factors for 90-day mortality. After the first step of multivariable analysis, only bis40map50_dur was statistically significant (*P* = .046) among BIS or MAP derived variables. In the final multivariable analysis, male sex, dysrhythmia, hemoglobin levels, albumin levels, and bis40map50_dur [odds ratio (OR), 1.26; *P* = .019] were associated with 90-day mortality (Table 2). Hosmer and Lemeshow goodness of fit test is not significant at 5% (*P* = .927).

**Table 2:** Association between variables and 90-day postoperative mortality

Variables	Univariable association			Multivariable association		
	OR	95% CI	*P*-value^a^	OR	95% CI	*P*-value^b^
Age (year)	1.02	0.99–1.06	.146			.438
Male sex^b^	2.64	1.07–6.55	.036	3.22	1.24–8.36	.017
Body mass index (kg/m^2^)	0.97	0.87–1.08	.539			
Category of surgery (vs. Stomach)			.239			
Colorectal	1.84	0.72–4.75	.205			
Hepatic	1.07	0.21–5.34	.938			
Biliary-pancreas	0.66	0.18–2.34	.518			
Volatile agent compared with TIVA	1.00	0.47–2.12	.998			
Duration of anesthesia (min)	1.00	0.99–1.00	.774			
bis40_dur (min)	1.00	0.99–1.01	.876			
bis40_5min	2.58	0.35–19.12	.353			
bis20_dur (min)	0.57	0.14–2.33	.432			
bis20_5min	< 0.01		.998			
eegsup_dur (min)	0.95	0.82–1.10	.483			
eegsup_5min	0.47	0.06–3.51	.465			
bis40map50_dur^b^ (min)	1.38	1.16–1.65	<.001	1.26	1.04–1.53	.019
bis20map50_dur (min)	1.89	0.44–8.08	.393			
map50_dur^*^ (min)	1.12	1.00–1.26	.057			
Past medical history						
Aortic stenosis	< 0.01		.999			
Congestive heart failure	< 0.01		.999			
Coronary artery disease	1.39	0.33–5.95	.655			
Hypertension	0.91	0.42–1.98	.809			
Peripheral vascular occlusive disease	< 0.01		.999			
Dysrhythmia^b^	5.20	1.75–15.47	.003	4.26	1.24–14.59	.021
Chronic obstructive pulmonary disease	3.13	0.72–13.60	.128			.805
Pulmonary hypertension	11.28	1.31–96.95	.027			.200
Stroke	< 0.01		.997			
Malignancy	4.77	1.13–20.16	.034			.219
Diabetes	2.07	0.95–4.52	.068			.618
Sleep apnea	< 0.01		.999			
Current smoker	1.74	0.70–4.32	.236			
Regular alcohol ingestion	0.86	0.35–2.13	.738			
ASA (compared with ASA I)			.012			.539
II	7.04	0.94–52.91	.058			.986
III	19.32	2.43–153.44	.005			.335
IV	< 0.01		.999			.795
Hemoglobin^b^ level (g/dL)	0.56	0.46–0.69	<.001	0.70	0.55–0.89	.004
Albumin^b^ level (g/dL)	0.11	0.05–0.21	<.001	0.21	0.09–0.47	<.001

^a^After binary logistic regression in ‘enter’ method, variables yielding *P*-values under 0.2 were regarded to be potential risk factors. ^b^In the final multivariable logistic regression analysis in ‘backward LR’ method, variables remaining in final model were regarded as significant risk factors. ^*^BIS or MAP derived variables not yielding *P*-values under 0.05 in the first step of multivariable analysis were excluded from final analysis

Abbreviations: *OR* Odds ratio; *CI* Confidence interval; *TIVA* Total intravenous anesthesia; *BIS* Bispectral index; bis40_dur, cumulative time in which BIS < 40; bis40_5min, incidence in which cumulative time of BIS < 40 lasted > 5 min; bis20_dur, cumulative time in which BIS < 20; bis20_5min, incidence in which cumulative time of BIS < 20 lasted > 5 min; *EEG* Electroencephalogram; eegsup_dur, cumulative time in which patient’s EEG was suppressed; eegsup_5min, incidence in which cumulative time of EEG suppression lasted > 5 min; *MAP* Mean arterial pressure; bis40map50_dur, cumulative time that BIS < 40 and MAP < 50 mmHg simultaneously; bis20map50_dur, cumulative time that BIS < 20 and MAP < 50 mmHg simultaneously; map50_dur, cumulative time that MAP < 50 mmHg; *ASA* The American Society of Anesthesiologists physical status

There were no significant differences of mean propofol concentration [2.84 μg/mL vs. 3.09 μg/mL respectively; 95% confidence interval (CI) -0.67 to 1.16; *P* = .597] and MAC of volatile anesthetics (0.91 vol% vs. 0.96 vol% respectively; *P* = .292) between patients with and without 90-day mortality.

### 180-day mortality

In univariable analysis, age, male sex, body mass index, category of operation, dysrhythmia, pulmonary hypertension, malignancy, diabetes, ASA classification, hemoglobin levels, albumin levels and bis40map50_dur were found to be potential risk factors for 180-day mortality. In multivariable analysis, category of surgical procedures, dysrhythmia, malignancy, ASA classification, hemoglobin levels and albumin levels were found to significantly predict 180-day mortality. No BIS or MAP derived variables had any significant relationship with 180-day mortality (Table 3). Hosmer and Lemeshow goodness of fit test is not significant at 5% (*P* = .326).

**Table 3:** Association between variables and 180-day postoperative mortality

Variables	Univariable association			Multivariable association		
	OR	95% CI	*P*-value^a^	OR	95% CI	*P*-value^b^
Age (year)	1.03	1.01–1.06	.007			.358
Male sex	1.52	0.87–2.64	.141			.243
Body mass index (kg/m^2^)	0.86	0.79–9.34	<.001			.230
Category of surgery^b^ (vs. Stomach)			.007			.008
Colorectal^b^	2.75	1.31–5.77	.008	3.34	1.51–7.39	.003
Hepatic	2.18	0.76–6.21	.146	1.90	0.63–5.72	.260
Biliary-pancreas	0.99	0.39–2.52	.983	1.23	0.46–3.30	.678
Volatile agent compared with TIVA	0.83	0.50–1.50	.492			
Duration of anesthesia (min)	1.00	0.99–1.00	.718			
bis40_dur (min)	1.00	0.99–1.00	.876			
bis40_5min	1.31	0.47–3.67	.605			
bis20_dur (min)	0.99	0.90–1.09	.791			
bis20_5min	1.88	0.44–8.04	.393			
eegsup_dur (min)	1.01	0.97–1.04	.745			
eegsup_5min	1.20	0.47–3.06	.700			
bis40map50_dur (min)	1.22	1.03–1.44	.018			.767
bis20map50_dur (min)	1.45	0.33–6.25	.622			
map50_dur (min)	1.05	0.93–1.18	.452			
Past medical history						
Aortic stenosis	3.44	0.43–27.58	.245			
Congestive heart failure	< 0.01		.999			
Coronary artery disease	0.62	0.15–2.59	.516			
Hypertension	1.21	0.72–2.05	.474			
Peripheral vascular occlusive disease	< 0.01		.999			
Dysrhythmia^b^	4.36	1.89–10.04	.001	3.43	1.31–9.00	.012
Chronic obstructive pulmonary disease	1.40	0.33–5.93	.645			
Pulmonary hypertension	5.16	0.61–43.59	.131			.563
Stroke	0.43	0.06–3.17	.410			
Malignancy^b^	5.13	1.85–14.23	.002	3.08	1.05–9.03	.040
Diabetes	2.09	1.21–3.61	.008			.540
Sleep apnea	< 0.01		.999			
Current smoker	1.14	0.55–2.35	.723			
Regular alcohol ingestion	0.71	0.37–1.39	.320			
ASA (compared with ASA I)			<.001			.055
II	7.55	1.81–31.41	.005	3.17	0.73–13.70	.122
III^b^	21.35	4.93–92.53	<.001	6.21	1.36–28.31	.018
IV	< 0.01		.999	0.00	0.00	.999
Hemoglobin^b^ level (g/dL)	0.56	0.48–0.65	<.001	0.79	0.66–0.95	.014
Albumin^b^ level (g/dL)	0.11	0.07–0.18	<.001	0.18	0.10–0.32	.000

^a^After binary logistic regression in ‘enter’ method, variables yielding *P*-values under 0.2 were regarded to be potential risk factors. ^b^In the final multivariable logistic regression analysis in ‘backward LR’ method, variables remaining in final model were regarded as significant risk factors

Abbreviations: *OR* Odds ratio; *CI* Confidence interval; *TIVA* Total intravenous anesthesia; *BIS* Bispectral index; bis40_dur, cumulative time in which BIS < 40; bis40_5min, incidence in which cumulative time of BIS < 40 lasted > 5 min; bis20_dur, cumulative time in which BIS < 20; bis20_5min, incidence in which cumulative time of BIS < 20 lasted > 5 min; *EEG* Electroencephalogram; eegsup_dur, cumulative time in which patient’s EEG was suppressed; eegsup_5min, incidence in which cumulative time of EEG suppression lasted > 5 min; *MAP* Mean arterial pressure; bis40map50_dur, cumulative time that BIS < 40 and MAP < 50 mmHg simultaneously; bis20map50_dur, cumulative time that BIS < 20 and MAP < 50 mmHg simultaneously; map50_dur, cumulative time that MAP < 50 mmHg; *ASA* The American Society of Anesthesiologists physical status

There were no significant differences of mean propofol dose (2.94 μg/mL vs. 3.09 μg/mL respectively; 95% CI − 0.46 to 0.75; *P* = .646) and MAC of volatile anesthetics (0.92 vol% vs. 0.96 vol% respectively; 95% CI − 0.01 to 0.09; *P* = .115) between patients with and without 180-day mortality.

### Subgroup analysis

There was no significant difference of mean propofol concentration (3.20 μg/mL vs. 3.04 μg/mL respectively; 95% CI − 0.61 to 0.29; *P* = .480) between patients who presented and who didn’t present double low (BIS < 40 and MAP < 50) among the total intravenous anesthesia (TIVA) cases. On the other hand, MAC of volatile anesthetics was higher in the patients who presented double low than who didn’t present double low (0.97 vol% vs. 0.95 V % respectively; 95% CI 0.01 to 0.41; *P* = .010) among the inhalation anesthesia cases.

Of the 659 patients who presented double low, ten patients were died in postoperative 90-day. Their mean duration of double low were 3.16 min (SD 4.57), while the mean duration of double low were 0.91 min (SD 1.22) in patients without 90-day mortality. There was significant difference of double low duration between these patients (*P* = .020).

## Discussion

The major finding of this study was that the duration of BIS values below 40 coupled with MAP less than 50 mmHg was associated with 90-day postoperative mortality, not 180-day postoperative mortality. This suggests that excessive anesthetic-induced brain suppression as well as intraoperative hypotension may be associated with adverse postoperative outcome.

Several early studies proposed statistical relationship between low BIS value (< 40 or 45) and postoperative mortality [4–6]. However, the cumulative duration of BIS value less than 40 or 20, and EEG suppression alone were not related to postoperative mortality in this study, consistent with previous further observational studies, randomized controlled trial, and meta-analysis [7, 9, 12, 17, 18]. In contrast, other previous studies showed the association between postoperative mortality and ‘double low’ of BIS and MAP, [9, 12–14] similar to our results. These findings may imply that it is not possible to predict mortality and adverse outcomes by excessive suppression alone, but only with combined hypotension.

Two meta-analyses proposed relationship between low BIS value alone and 90-day or 1- year postoperative mortality, not 30-day postoperative mortality [19, 20]. On the other hand, other previous studies reported that double low was associated with 30-day postoperative mortality [9, 13] or 90-day postoperative mortality [12, 14]. This finding suggests that intraoperative low BIS values and blood pressure seem to be related to early-to-intermediate postoperative mortality and not to intermediate-to-long term mortality. The sequelae of intraoperative events and excessive anesthesia can lead to early postoperative complications which is associated with early-to-intermediate mortality, but the effect seems to be time-limited.

In this study, mean propofol concentrations were not statistically different between patients with or without double low among TIVA cases. Therefore, patients who presented double low may have had higher anesthetic vulnerability, which means that some patients are prone to show lower BIS values and hypotension in similar anesthetic dosage, followed by postoperative adverse outcomes. On the other hand, in inhalational anesthesia, mean MAC of volatile anesthetics was higher in the patients with double low. In this respect, excessive anesthesia also can be a cause of double low and, furthermore, postoperative mortality. In addition, as Charier and colleagues mentioned in their review, opioid administration can affect both hypnosis and arterial hypotension, [21] so that it would be more pragmatic to take into account the nociception-analgesia balance as well. Further research is needed to investigate the difference in anesthetic vulnerability according to the type of anesthesia or opioid administration. Nevertheless, BIS monitoring and titration of anesthetics can help avoid unnecessarily deep anesthesia and possible neurotoxic effects in vulnerable patients, [22] yet there is still a lack of evidence by prospective studies [14] whether avoiding ‘double low’ state can improve postoperative outcomes.

The Vital Recorder program, which was used to collect BIS values, suppression ratios and MAP data in this study, is an automatic recording device for obtaining high-resolution time-synchronized physiological data from multiple anesthesia devices [23]. With this software we could obtain stored digitalized data for every patient, as well as accurately compute the independent variables related to BIS and MAP. Furthermore, intraoperative target site propofol concentrations in TIVA and MAC of volatile agents in inhalational anesthesia were recorded in real time.

This study derived relationship between the cumulative duration of concurrent double low and postoperative mortality, especially focusing on the abdominal surgeries including gastrointestinal tract, liver, biliary tract, and pancreas surgeries. Other previous studies revealed comparable results on influence of low BIS and hypotension in other types of surgeries, [13, 14] and this study can support the results so far in respect of major abdominal surgery. Meanwhile, the cutoff value of MAP was 50 mmHg in this study according to several definitions of clinically significant hypotension [12, 15, 24, 25]. Although it would be less conservative than the cutoff value of 75 mmHg, we tried to investigate narrower sense of definition of double low.

This study had several limitations. First, the incidence of postoperative mortality was relatively smaller than that in previous reports [10, 12, 26]. For a more accurate statistical analysis, a larger number of cases would be needed. Second, this study has limitation from the design of retrospective study. Data can be incomplete. Nevertheless, all intraoperative data were completely obtained using the ‘Vital Recorder' program. Third, we included only patients receiving major abdominal surgeries to decrease other bias originated from different surgery and population. However, this may be another limitation for generalization of our results.

## Conclusions

In conclusion, the cumulative duration of BIS values less than 40 coupled with MAP less than 50 mmHg was associated with 90-day postoperative mortality, not 180-day postoperative mortality.

## Data Availability

All original datasets used and analyzed during the current study are available from the corresponding author on reasonable request.

## Abbreviations

BIS: Bispectral index
MAP: Mean arterial pressure
MAC: Minimal alveolar concentration
EEG: Electroencephalography
bis40_dur: Cumulative time in which BIS values were less than 40
bis40_5min: Incidence in which cumulative time of BIS values less than 40 lasted more than 5 min
bis20_dur: Cumulative time in which BIS values were less than 20
bis20_5min: Incidence in which cumulative time of BIS values less than 20 lasted more than 5 min
eegsup_dur: Cumulative time in which patient’s EEG was suppressed
eegsup_5min: Incidence in which cumulative time of EEG suppression lasted more than 5 min
map50_dur: Cumulative time that MAP was less than 50 mmHg
bis40map50_dur: Cumulative time that BIS values were less than 40 and MAP was less than 50 mmHg simultaneously
bis20map50_dur: Cumulative time that BIS values were less than 20 and MAP was less than 50 mmHg simultaneously
ASA: American Society of Anesthesiologists
VIF: Variance inflation factor
TIVA: Total intravenous anesthesia
